# The impact of COVID-19 on sexual behavior, HIV prevention interest, general healthcare access, and other HIV risk factors among trial participants in Malawi, South Africa, Uganda, and Zimbabwe

**DOI:** 10.3389/frph.2023.1270419

**Published:** 2023-10-30

**Authors:** Noah Mancuso, Florence Mathebula, Miria Chitukuta, Kudzai V. Matambanadzo, Siyanda Tenza, Krishnaveni Reddy, Lumka Nobula, Doreen Kemigisha, Marie C. D. Stoner

**Affiliations:** ^1^ Women's Global Health Imperative, RTI International, Atlanta, GA, United States; ^2^ Wits RHI, University of the Witwatersrand, Johannesburg, South Africa; ^3^ Clinical Trials Research Centre, University of Zimbabwe, Harare, Zimbabwe; ^4^ Desmond Tutu HIV Foundation, Cape Town, South Africa; ^5^ Makerere University—Johns Hopkins University (MU-JHU) Research Collaboration, Kampala, Uganda; ^6^ Women's Global Health Impeative, RTI International, Berkeley, CA, United States

**Keywords:** HIV prevention, COVID-19, adolescent girls and young women (AGYW), pregnant, breastfeeding, clinical trial, dapivirine vaginal ring, oral PrEP

## Abstract

**Introduction:**

The COVID-19 pandemic greatly impacted HIV prevention and care globally. The pandemic also had disproportionate impacts on the financial, emotional, and physical wellbeing of women and girls in East and Southern Africa, who were already at increased HIV vulnerability. This study aimed to understand how the COVID-19 pandemic and its response efforts impacted the sexual behavior, HIV prevention interest, general healthcare access, and other HIV risk factors of women and girls in HIV prevention studies.

**Methods:**

Using the socio-ecological model (SEM), an explanatory sequential mixed-methods analysis was performed with data from four Microbicide Trial Network (MTN) studies on different populations—adolescent girls and young women (AGYW), pregnant persons, breastfeeding persons, and couples—in Malawi, South Africa, Uganda, and Zimbabwe. Descriptive statistics for outcomes of interest were calculated within each study separately and Chi-squared tests of independence were performed to evaluate associations between study population and outcomes. Excerpts from study qualitative interviews were stratified into code reports which were then summarized into memos with key themes and considerations of the SEM framework to provide context to quantitative findings.

**Results:**

Few participants (8/731) had known or suspected COVID-19 infection. Sexual frequency and alcohol use decreased most often among AGYW compared to pregnant or breastfeeding women and couples (*p*-value < 0.001). The pandemic had little impact on changes in reported HIV prevention interest or access to HIV prevention study products. Healthcare access was impacted for everyone, with couples most likely to report decreases in access (*p*-value < 0.001). From qualitative interviews, economic instability, adverse mental health, and increased violence due to COVID-19 caused increased strain on other factors related to HIV vulnerability.

**Conclusions:**

While interest in HIV prevention did not change and a few HIV risks decreased for most women and girls, other vulnerabilities to HIV increased due to the COVID-19 pandemic, highlighting the importance of continued access to HIV prevention for women and girls. More research is needed to better understand the long-term impact of COVID-19 on HIV prevention and vulnerability in community populations.

## 1. Introduction

The emergence of coronavirus disease 2019 (COVID-19) and the policy response to mitigate spread of the causative agent, severe acute respiratory syndrome coronavirus 2 (SARS-CoV-2), has had far-reaching impact on people’s health and well-being globally. In particular, access to healthcare services dropped significantly after 2020 due to the implementation of lockdown policies and shelter-in-place orders, disruption to public transportation services, and because individuals were less likely to seek healthcare services out of a fear of getting infected with SARS-CoV-2 (1). In many countries in East and Southern Africa, which have a high burden of HIV, access to HIV prevention and care services was greatly disrupted during the pandemic (1, 2). In an analysis of 24 countries in Africa, HIV referrals and HIV testing declined over 40% from 2019 to 2020 (1). A systematic review evaluating the impact of the COVID-19 pandemic on accessing HIV services in South Africa found decreases in HIV testing, in positive HIV tests, and in initiation of antiretroviral therapy (ART); effects of the pandemic were greater at public health facilities compared to private (3). Additionally, prevention of mother-to child transmission (PMTCT) services and other HIV prevention methods declined across Africa (1). These disruptions in care caused by COVID-19 likely had the greatest impact among those most vulnerable to HIV in East and Southern Africa such as adolescent girls and young women (AGYW), pregnant persons, and breastfeeding persons, though little has been published about how these disruptions affected access to HIV prevention services across these different populations.

Women and girls bore the brunt of much of the COVID pandemic (economically, mentally, physically) and are also at high risk for HIV in East and Southern Africa (4). In 2021, women and girls accounted for 63% of all new HIV infections with a disproportionate number of infections among AGYW compared to young men of the same age (2). In addition, the COVID-19 pandemic has compounded existing vulnerabilities faced by women and girls such as reductions in paid work, increased mental and emotional stress, and increased rates of gender-based violence (4, 5). AGYW were more likely to spend additional hours on domestic work during the pandemic compared to boys of the same age, and experienced increased child marriage and school dropout (6). Additionally, access to antenatal care first visits fell by 5% for pregnant persons across Africa in 2020 relative to 2019, and age-under-5 consultations decreased by 23% (1). Pregnancy and postpartum periods are a time of increased risk of HIV acquisition due to biological and socio-contextual reasons (7, 8). The increased vulnerability to HIV for women and girls in this region compounded by the effects of the COVID-19 pandemic highlight the importance of sustained access to care during this time.

The objective of our mixed methods study was to assess how the emergence of and response to COVID-19 impacted sexual behavior, HIV prevention interest, general healthcare access, and other HIV risk factors among women and girls in Malawi, South Africa, Uganda, and Zimbabwe. We used data from four distinct HIV prevention trial populations including AGYW [Microbicide Trials Network (MTN)-034/REACH], pregnant persons (MTN-042/DELIVER), breastfeeding persons (MTN-043 study/B-PROTECTED), and couples where female participants discussed HIV prevention options with their partners (MTN-045/CUPID). We used quantitative data to examine changes in sexual behavior, interest in preventing HIV, access to healthcare services, mental health, experiences with violence, and access to resources like money and food across the four study populations during the pandemic. Qualitative data was used to contextualize quantitative findings and any differences across populations.

## 2. Methods

The Microbicide Trials Network (MTN) is a collaborative clinical trials network focused on developing and testing interventions that prevent the transmission of HIV, including new biomedical HIV prevention products such as the dapivirine vaginal ring. MTN is funded by the National Institute of Allergy and Infectious Diseases (UM1AI068633, UM1AI068615, UM1AI106707), with co-funding from the Eunice Kennedy Shriver National Institute of Child Health and Human Development and the National Institute of Mental Health, all components of the U.S. National Institutes of Health (NIH). The dapivirine vaginal ring (“ring”) used in MTN studies was developed and supplied by the International Partnership for Microbicides (IPM). Oral tenofovir disoproxil fumarate/emtricitabine (TDF/FTC) PrEP (“pills”) used in MTN studies were donated by Gilead Sciences.

### 2.1. Study setting and participants

The present study included data from participants across four separate studies at six sites in Malawi, South Africa, Uganda, and Zimbabwe. Each site has long-established research networks and active community engagement teams that recruited participants from a variety of primary care health clinics, family planning clinics, HIV testing facilities, and other community locations across urban, peri-urban, and rural communities. Eligible participants in this study were HIV negative at study start, were enrolled in one of the four MTN studies and answered COVID-19 questions that were incorporated into the study protocols during the pandemic. This sub-sample of MTN participants represents approximately 75% of the total population across the four MTN studies. Each MTN study is briefly described below to provide context into the respective study setting; additional details about the design and populations have been published previously.

#### 2.1.1. MTN parent studies

The MTN-034/REACH study was a crossover trial aimed at collecting information about the safety of, acceptability of, and preferences for the monthly ring and daily oral PrEP pills among AGYW. The study was conducted between January 2019 and September 2021 in Johannesburg and Cape Town, South Africa; Kampala, Uganda; and Harare, Zimbabwe (9, 10). The MTN-042/DELIVER study was a randomized, open-label trial aimed at assessing the safety, adherence, and acceptability of the ring and pills when used during pregnancy. The study was conducted among women 8–9 months pregnant at four sites: Blantyre, Malawi; Johannesburg, South Africa; Kampala, Uganda; and Chitungwiza, Zimbabwe (11, 12). The MTN-043/B-PROTECTED study was a randomized, open-label, mother-infant pair trial aimed at assessing the safety, adherence, and acceptability of the ring and pills when used during breastfeeding. The study was conducted between September 2020 and November 2021 at four sites: Blantyre, Malawi; Johannesburg, South Africa; Kampala, Uganda; and Chitungwiza, Zimbabwe (13, 14). The MTN-045/CUPID study was a cross-sectional study aimed at assessing hypothetical preferences related to dual-purpose prevention products (ring, pills, vaginal insert, and vaginal film) that could be used to prevent unintended pregnancies and HIV infection among couples. The study was conducted between November 2019 and December 2020 at two sites: Kampala, Uganda and Harare, Zimbabwe (15, 16).

### 2.2. Quantitative data collection

Quantitative data about demographics and the impact of COVID-19 were collected via self-report at study enrollment across all four studies. Demographic data included information about the participant’s age, partner status, and income. Data collected on COVID-19 included the impact of the pandemic on each of the following: mental health; feelings of connection to primary partners, family, and friends; sexual behavior; access to healthcare; and interest in HIV prevention. The COVID-19 questionnaire was standardized to ensure that the same questions were asked across all four studies.

### 2.3. Qualitative data collection

Qualitative data were collected during 60–90 min in-depth interviews and focus group discussions at pre-specified time points throughout each study. Interviews were conducted by trained social scientists in local languages using semi-structured interview guides. Interviewers asked participants amongst other questions, how COVID-19 had affected their overall health, relationships with others, access to resources, and their experiences with both HIV prevention products and study participation. All interviews were transcribed, translated to English, and then reviewed internally at the study sites before going through quality control by staff at the qualitative data management center at RTI International.

### 2.4. Analysis

We used an explanatory sequential mixed methods design whereby we first analyzed quantitative data and then looked to the qualitative data to help contextualize the quantitative results (18). The socio-ecological model (SEM)—which is used to conceptualize the complex interplay of individual, interpersonal, community, and societal factors that impact health—was used to help understand the many factors related to COVID-19 and its response that impacted participants (17). The SEM was used in the organization of the quantitative results and during the analysis of qualitative code reports. Discussions about the SEM helped structure and guide the discussion about study findings and interpretation of the results.

#### 2.4.1. Quantitative analysis

Using quantitative data across all four MTN studies, we calculated descriptive statistics for participant demographics and known or suspected COVID-19 infection of participants and people they know. We then calculated descriptive statistics for our outcomes of interest (e.g., sexual behavior, HIV prevention interest, healthcare access, and other HIV risk factors). All descriptive statistics were calculated within each study separately; however, some response levels were combined within a study for more consistent response levels and clearer comparisons across study populations. We then performed Chi-squared tests of independence, with frequency weighting based on size of study population, to evaluate associations between study population and outcomes of interest. All statistical analyses were conducted using RStudio v2022.12.0 (Rstudio Team, Boston, MA, USA).

#### 2.4.2. Qualitative analysis

Interview transcripts had previously been double coded by a team of experienced analysts at the qualitative data management center using pre-specified codes. Coded excerpt reports were exported on a regular basis to test intercoder reliability and come to consensus on discrepancies. The two lead authors and three qualitative analysts from the research sites reviewed all excerpts coded with a COVID-19 code (*n* = 1,094 excerpts). These excerpts stratified by MTN parent study and then sorted into code reports based on co-codes with at least 10 excerpts (those with less than 10 were grouped into an “Other” report). All excerpts were analyzed using Dedoose software v9.0.17 (Socio Cultural Research Consultants LLC, Los Angeles, CA, USA). Code reports were grouped based on level of SEM (e.g., individual, interpersonal, etc.) and summary memos were created. Each summary memo included key themes and considerations for how these themes fit into the SEM framework and to which quantitative data they provided context. Analysts met every 3 weeks during the memo writing process to discuss key findings, come to consensus about themes, and relate these back to the quantitative results.

### 2.5. Ethics statement/ethical considerations

Each study protocol was approved by the Institutional Review Board or Ethics Committee at each site in the respective study. The four studies were overseen by the regulatory infrastructure of the Division of AIDS and the Microbicide Trials Network. Participants in all four MTN studies provided written consent (and assent accompanied by parental consent for minors where applicable) prior to participation in their respective study and provided verbal consent prior to participation in qualitative activities. Quotes are labeled with pseudonyms to protect participants’ identities.

## 3. Results

### 3.1. Participant characteristics

Nearly half of the 731 participants (45%) resided in Zimbabwe and most (85%) were female; male participants were included only in couples in the MTN-045 study (Table 1). The median age for MTN-034 participants (18 years) was younger than the other three studies (MTN-042 = 25 years; MTN-043 = 25 years; MTN-045 = 28 years), as this study was focused on AGYW. Most participants (96%) reported having a primary partner. Of those with a partner, more than 1 in 5 reported that they knew or suspected their partner of having other partners. Participants from MTN-045 with couples were more likely to report having their own source of income compared to participants in MTN-034 who were AGYW (69% vs. 13%). Few participants reported having known or suspected COVID-19 infection (1%) and most reported not knowing anyone else who was infected with COVID-19 (73%).

**Table 1:** Demographics from participants in four MTN studies, 2019 to 2021 in Malawi, South Africa, Uganda, and Zimbabwe.

		MTN-034/REACH	MTN-042/DELIVER	MTN-043/B-PROTECTED	MTN-045/CUPID
Population		AGYW n (%)	Pregnant Women n (%)	Breastfeeding Women n (%)	Couples n (%)
Total		166	150	197	218
Years/Dates of Study		2019–2021	Cohort 1:2020–2021	2020–2021	2019–2020
Country of residence	Malawi	0 (0.0)	27 (18.0)	39 (19.8)	0 (0.0)
	South Africa	127 (76.5)	42 (28.0)	36 (19.3)	0 (0.0)
	Uganda	28 (16.9)	44 (29.3)	55 (27.9)	3 (1.4)
	Zimbabwe	11 (6.6)	37 (24.7)	67 (34.0)	215 (98.6)
Gender	Female	166 (100)	150 (100)	197 (100)	109 (50)
	Male	0 (0.0)	0 (0)	0 (0)	109 (50)
Age	Median (IQR)	18 (17,19)	25 (21, 28)	25 (23,30)	28 (24, 34)
Has primary partner/considers person in study to be primary partner	Yes	149 (89.8)	146 (97.3)	192 (97.5)	216 (99.1)
	No	17 (10.2)	4 (2.7)	5 (2.5)	2 (0.9)
Partner has other partners^a^	Yes, I know or I suspect	31 (18.7)	42 (28.8)	63 (32.8)	22 (10.1)
	No	44 (26.5)	37 (25.3)	43 (22.4)	196 (89.9)
	Don’t know	74 (44.6)	67 (45.9)	86 (44.8)	NA
Own source of income	Yes	22 (13.3)			553 (69.1)
	No	144 (86.7)			247 (30.9)
Known or suspected infection w/COVID-19	Yes, confirmed	0 (0.0)	0 (0)	2 (1.3)	0 (0)
	Yes, suspected	1 (0.6)	0 (0)	1 (0.6)	4 (1.8)
	Not sure	2 (1.2)	0 (0)	0 (0)	0 (0)
	No, tested negative	4 (2.4)	1 (0.8)	6 (3.9)	2 (0.9)
	No	159 (95.8)	129 (99.2)	147 (94.2)	213 (97.3)
# of people known to be infected w/COVID-19	0	112 (67.5)	111 (85.4)	123 (78.9)	184 (84.4)
	1 or more	54 (32.5)	19 (14.6)	33 (21.1)	34 (15.6)

^a^ Totals do not all add up to 100% as not all participants had current partners (except in MTN-045).

### 3.2. Mixed methods results

To explore our objectives, results are organized by themes that emerged in the quantitative data related to changes in sexual behavior, HIV prevention interest (general interest and study product interest), healthcare services access, and other HIV risk behaviors. Qualitative themes that emerged within each of these four quantitative themes are presented for context and to add further explanation for quantitative results.

#### 3.2.1. Sexual behavior

Reported changes in the frequency of sex due to COVID-19 differed across study populations (*χ*^2^ = 69.65, *p*-value < 0.001). Couples reported increases in the frequency of sex (MTN-045 = 22%) much more than AGYW, pregnant, and breastfeeding women (MTN-034 = 5%; MTN-042 = 7%; MTN-043 = 3%). More AGYW reported decreases in the frequency of sex (MTN-034 = 37%) compared to pregnant and breastfeeding women (MTN-042 = 18%; MTN-043 = 24%) and couples (MTN-045 = 13%) (Table 2).

**Table 2:** The impact of COVID-19 on different areas of clinical trial participants’ lives across four MTN studies.

		MTN-034/REACH	MTN-042/ DELIVER	MTN-043/B- PROTECTED	MTN-045/CUPID	Chi-square Test
		AGYW n (%)	Pregnant Women n (%)	Breastfeeding Women n (%)	Couples n (%)	
How has COVID-19 impacted…						
Level of anxiety	Decreased	8 (4.8)	3 (3.0)	15 (9.6)		*χ*^2^ = 5.52 *p-value* = 0.24
	Increased	68 (41.0)	40 (40.4)	62 (39.7)		
	Not changed	90 (54.2)	56 (56.6)	79 (50.6)		
Feelings of depression	Decreased	16 (9.6)	4 (4.1)	17 (10.9)		*χ*^2^ = 4.12 *p-value* = 0.39
	Increased	42 (25.3)	23 (23.5)	38 (24.4)		
	Not changed	108 (65.1)	71 (72.4)	101 (64.7)		
Feelings of connection to family	Decreased	37 (22.3)	14 (14.1)	29 (18.6)	93 (42.5)	*χ*^2^ = 58.13 *p-value* < 0.001
	Increased	39 (23.5)	13 (13.1)	23 (14.7)	44 (20.1)	
	Not changed	90 (54.2)	72 (72.8)	104 (66.7)	82 (37.4)	
Feelings of connection to friends	Decreased	70 (42.2)	34 (34.3)	52 (33.3)	139 (63.5)	*χ*^2^ = 50.17 *p-value* < 0.001
	Increased	21 (12.7)	10 (10.1)	11 (7.1)	18 (8.2)	
	Not changed	75 (45.2)	55 (55.5)	93 (59.6)	62 (28.3)	
Feelings of connection to primary partner^a^	Decreased	22 (13.3)	10 (10.1)	16 (10.3)	26 (11.9)	*χ*^2^ = 12.84 *p-value* = 0.04
	Increased	17 (10.2)	9 (9.1)	11 (7.1)	42 (19.2)	
	Not changed	113 (68.1)	65 (65.7)	117 (75.0)	151 (69.0)	
Frequency of sex	Decreased	62 (37.3)	18 (18.4)	37 (23.7)	29 (13.2)	*χ*^2^ = 69.65 *p-value* < 0.001
	Increased	8 (4.8)	7 (7.1)	5 (3.2)	48 (21.9)	
	Not changed	96 (57.8)	73 (74.5)	114 (73.1)	142 (64.8)	
Frequency of using condoms during sex	Decreased	14 (8.4)	7 (7.1)	9 (5.8)	18 (8.2)	*χ*^2^ = 11.89 *p-value* = 0.06
	Increased	16 (9.6)	4 (4.1)	4 (2.6)	8 (3.6)	
	Not changed	136 (81.9)	88 (88.8)	143 (91.7)	193 (88.1)	
Access to condoms	Decreased	4 (2.4)	7 (7.1)	10 (6.4)		*χ*^2^ = 5.68 *p-value* = 0.23
	Increased	10 (6.1)	3 (3.0)	5 (3.2)		
	Not changed	151 (91.5)	89 (89.9)	141 (90.4)		
Access to health care	Decreased	20 (12.0)	26 (26.3)	42 (26.9)	93 (42.5)	*χ*^2^ = 49.34 *p-value* < 0.001
	Increased	16 (9.6)	11 (11.1)	11 (7.1)	6 (2.7)	
	Not changed	130 (78.3)	62 (62.6)	103 (66.0)	120 (54.8)	
Access to money for necessary items	Decreased	74 (45.1)	55 (55.5)	98 (62.8)	168 (76.7)	*χ*^2^ = 52.05 *p-value* < 0.001
	Increased	11 (6.7)	9 (9.1)	14 (9.0)	18 (8.2)	
	Not changed	79 (48.2)	35 (35.3)	44 (28.2)	33 (15.1)	
Amount of food eaten	Decreased	35 (21.1)	39 (39.4)	79 (50.6)	142 (64.8)	*χ*^2^ = 104.92 *p-value* < 0.001
	Increased	46 (27.7)	7 (7.1)	9 (5.8)	13 (5.9)	
	Not changed	85 (51.2)	53 (53.5)	68 (43.6)	64 (29.2)	
Alcohol consumption	Decreased	56 (33.7)	1 (1.0)	4 (2.6)	35 (16.0)	*χ*^2^ = 92.49 *p-value* < 0.001
	Increased	4 (2.4)	0 (0)	0 (0)	9 (4.1)	
	Not changed	106 (63.9)	98 (99.0)	152 (97.4)	175 (79.9)	
Violence in household	Decreased	15 (9.1)	2 (2.0)	12 (7.7)	17 (7.8)	*χ*^2^ = 7.48 *p-value* = 0.28
	Increased	12 (7.3)	6 (6.1)	14 (9.0)	11 (5.0)	
	Not changed	138 (83.6)	90 (91.8)	130 (83.3)	191 (87.2)	

^a^ Totals do not all add up to 100% as not all participants had current partners (except in MTN-045).

In the qualitative data, lockdowns and travel restrictions due to COVID-19 affected the frequency in which participants had sex. For couples, lockdowns and travel restrictions kept them at home together more than before which often resulted in increases in the frequency of sex with each other and some participants reported reductions in infidelity in relationships.

*“He used to be an outgoing person, he would go out and drink at night and find another lady and we will fight about that, so since COVID-19 [outbreak] everything changed. He does not have money to go out… since there is lockdown so there is a curfew so he will be home most of the time, so no arguments between us about going out and stuff. There is harmony from my side… I think he also feels there is more harmony, there is more family time, so I think we are fine [giggles].”* (MTN-042, Female, IDI, Johannesburg, South Africa).

Among participants who were not living with a primary partner, travel restrictions from COVID-19 and permit requirements to leave the home made it harder to visit each other. This resulted in less frequent sex and was mostly reported by AGYW:

*“I only have one partner and it-just-just because of the non-visitations during level 5 and of course you had to have a permit or reason to be leaving your home, so… I couldn’t actually do any visitations.”* (MTN-034, Female, 19 years old, SIDI, Johannesburg, South Africa).

Financial instability brought about by COVID-19 and its response also impacted the sexual behavior of participants, however in different ways. A few women across each study reported that the financial impact of COVID-19 forced them to resort to sex work:

“*When the COVID-19 pandemic broke out, my husband left and I was left with nothing to eat… So, I decided to do sex work.”* (MTN-042, Female, IDI, Kampala, Uganda).

Other participants explained that the financial stress of the pandemic reduced their desire, or that of their partners, to have sex which resulted in less frequent sexual activity.

#### 3.2.2. HIV prevention interest

Most participants reported no influence of COVID-19 on their interest in preventing HIV, but this varied across study populations (*χ*^2^ = 14.84, *p*-value = 0.02) (Table 3). More AGYW and couples reported an increased interest in preventing HIV due to COVID-19 (27% for both) than pregnant and breastfeeding women (16%; 14%, respectively). Similarly, reported interest in using HIV prevention study products did not change for most participants due to COVID-19 (MTN-034 = 80%, MTN-042 = 83%; MTN-043 = 87%). More AGYW reported an increased interest in study products (18.1%) compared to pregnant women (11.1%) and breastfeeding women (9.0%).

**Table 3:** Reported concerns about HIV and COVID-19 and the impact of COVID-19 on interests in preventing HIV and using HIV prevention products among participant in four MTN trials.

		MTN-034/REACH	MTN-042/DELIVER	MTN-043/B-PROTECTED	MTN-045/CUPID	Chi-square Test
		AGYW n (%)	Pregnant Women n (%)	Breastfeeding Women n (%)	Couples n (%)	
Which concerns you more right now?	Getting COVID-19	67 (40.4)	80 (61.5)	72 (46.1)	64 (29.2)	*χ*^2^ = 110.75 *p-value* < 0.001
	Getting HIV	19 (11.4)	21 (16.2)	58 (37.2)	33 (15.1)	
	Both equally	63 (38.0)	24 (18.5)	21 (13.5)	110 (50.2)	
	Neither concerns me	17 (10.2)	5 (3.8)	5 (3.2)	12 (5.5)	
How has COVID-19 influenced your interest in preventing HIV?	Decrease	10 (6.0)	12 (9.2)	15 (9.6)	18 (8.2)	*χ*^2^ = 14.84 *p-value* = 0.02
	Increased	44 (26.5)	21 (16.2)	22 (14.1)	60 (27.4)	
	No influence	112 (67.5)	97 (74.6)	119 (76.3)	141 (64.4)	
Interest in study products?	Decreased	3 (1.8)	8 (6.3)	7 (5.5)		*χ*^2^ = 9.81 *p-value* = 0.04
	Increased	30 (18.1)	14 (11.1)	14 (9.0)		
	Not changed	133 (80.1)	104 (82.5)	135 (86.5)		

During qualitative interviews, participants reported few disruptions from COVID-19 in access to their HIV prevention study products despite experiencing significant disruptions in accessing other forms of healthcare services. This constant access to HIV prevention through participation in the studies helped maintain, and in some cases increase, their interest in continuing to use HIV prevention products. For a few participants, however, discreetly taking daily pills became a bigger issue than access during the pandemic. Some participants reported moving back in with family to save money, which made it difficult to hide pill use. One participant said of taking the pills with family around in the house:

"*I was not swallowing the tablet. I could not swallow them when there were people.”* (MTN-034, Female, FGD, Kampala, Uganda).

This was exacerbated when studies sent extra products (both pills and rings) home to reduce the number of in-person clinic visits, and participants had to find a way to discreetly store products where others in the home would not see them.

Some participants were able to use lockdown to their advantage in adhering to HIV prevention products, especially the pill, by creating habits of daily use. This was not always possible before COVID-19 because participants were travelling frequently for work and socialization. One woman said that:

*“When it’s lockdown, at least I drink the pills every day.”* However, when COVID-19 restrictions were lifted, she struggled to continue to adhere, “*Everything is allowed so other things are easily forgotten. So, I would go outside and sit with my friends and have fun or go out to drink if we didn’t go anywhere. We would go out and drink and I would forget about the pills.”* (MTN-034, Female, 19 years old, IDI, Cape Town, South Africa).

When weighing concerns for HIV compared to COVID-19, pregnant women (62%) were significantly more concerned for COVID-19 than HIV compared to AGYW, breastfeeding women, and couples (40%; 46%; 29%, respectively). In the qualitative interviews, this was attributed to familiarity with HIV and knowing that a treatment to prolong life exists. For COVID-19, participants spoke of the many unknowns for the disease and potential long-term side effects. This was especially concerning for pregnant women who were worried about the health impact of COVID-19 on their future child.

*“For my future health, mostly you know at this age, HIV is not like COVID-19, for HIV you can get some tablets and then your life goes on like that.”* (MTN-042, Female, IDI, Johannesburg, South Africa).

#### 3.2.3. Healthcare access

There were significant impacts on reported access to healthcare services due to COVID-19, which differed by study population (*χ*^2^ = 49.34, *p*-value < 0.001). About 40% of couples reported decreased access to healthcare services due to COVID-19, compared to roughly 26% of pregnant and breastfeeding women and only 12% of AGYW. In the qualitative interviews, participants reported difficulty accessing public clinics due to restricted hours, lack of transportation, and fear of contracting COVID at clinics as they were regarded to be hotspots. This resulted in some women changing their access of healthcare by visiting clinics less often and instead relying on home remedies or self-prescribing from a pharmacy.

*“I get worried a lot especially now there is COVID-19, because like… you have to come to the clinic, but you are not sure who has it* [COVID-19]*, who does not, yet you have to bring the baby to the clinic for the check-up.”* (MTN-042, Female, IDI, Johannesburg, South Africa).

For those who could access public clinics, there were frequent reports of negative experiences, like being yelled at, as well as long queues with shortages of child immunizations, staff, and products such as condoms.

*“COVID-19 impacted us a great deal because sometimes you would go to the clinic and not get the condoms. They* [healthcare workers] *would tell us that they ran out of stock, and we should come back the following day to check.”* (MTN-043, Female, IDI, Johannesburg, South Africa).

#### 3.2.4. Other HIV risk behaviors

About 2 in 5 participants reported increases in anxiety, while nearly 1 in 4 reported increases in depression, neither of which differed significantly by study population. In the qualitative interviews, these changes were frequently related to participants’ feelings of a lack of connection to others, which were impacted by COVID-19 because of restricted in-person activities—closing schools and churches—and travel restrictions limiting participants’ abilities to visit others for important events like weddings, birthdays, and funerals.

*“*[The] *issue of going to church, we had different sessions whereby only a few were allowed to pray, making it very difficult for us to be able to meet with good or best friends because of having these sessions. So, these things affected me so much*.” (MTN-043, Female, IDI, Blantyre, Malawi).

The closing of bars and clubs were reported to be drivers of reduced alcohol consumption, especially among AGYW whose alcohol consumption due to COVID-19 decreased significantly more than other populations (*χ*^2^ = 92.49, *p*-value < 0.001). As one participant explains,

“*Every week we would drink. But now that it is lockdown, we are not that much into alcohol.*” (MTN-034, Female, 19 years old, IDI, Cape Town, South Africa).

COVID-19 created financial strain for many participants and their families, which negatively impacted their access to food and experiences of violence and changed their family planning goals. Couples most frequently reported decreases in the amount of food eaten (65%), followed by breastfeeding women (51%), pregnant women (39%), and AGYW (21%). Though the MTN research studies and local governments provided some food assistance, a few participants spoke of the power imbalances and sexual coercion that this created during COVID-19:

“*During that time the government was giving out maize flour and the soldiers also wanted to buy* [sex from the commercial sex workers] *… if he falls in love with you, he could just tell you ‘Give me* [sex] *and I give you a box of milk.’*” (MTN-034, Female, 16 years old, Kampala, Uganda).

Experiences of violence largely did not change across study population, but a few women did report increases in violence due to COVID-19. This was often attributed to male partners being at home all day and stressed due to job loss and being unable to provide food or money for their family.

“*He was so protective and was so caring but… during the time COVID came in, he didn’t have money and we didn’t have food at home and yet I was also not working, and he changed a bit. He changed his ways and became so tough, yet he wasn’t like that before.”* (MTN-045, Female, IDI, Harare, Zimbabwe).

The individual financial strain of COVID-19 and its impact on the larger economy of many countries also impacted some couples’ family planning.

*“When we last had this conversation, we said because of the country’s economy we cannot continue bearing children. And, due to corona virus, money is scarce, so we cannot afford to buy preparation for the baby.”* (MTN-045, Male, IDI, Harare, Zimbabwe).

## 4. Discussion

In this mixed-methods study of the effects of COVID-19 on AGYW, pregnant women, breastfeeding women, and couples participating in four MTN HIV prevention studies, we found in quantitative surveys that most participants reported no influence from COVID-19 on their interest in preventing HIV and that their interest in using HIV prevention study products did not change. In qualitative interviews, we found that COVID-19 increased frequency of sex for those who lived with a partner but decreased sex for those who had to travel to visit partners, typically younger women. A few women across each study shared in interviews that the financial impact of COVID-19 forced them to resort to sex work or affected power dynamics negatively within relationships. Due to participation in their respective MTN studies, women reported in quantitative surveys that they had few disruptions from COVID-19 in accessing study HIV prevention products (ring and pill) despite experiencing significant disruptions in accessing other forms of healthcare services. This constant access to HIV prevention products helped maintain, and in some cases increased, their interest and adherence to HIV prevention products. However, some participants said in interviews that it was harder to be discreet and hide study products from others while spending more time at home during shelter-in-place ordinances.

Our results align with the SEM by highlighting the interplay of different levels of influence on HIV prevention during the COVID-19 pandemic. At the individual level, we found that access to HIV prevention methods remained consistent, although discretion in taking or hiding study product from others was difficult. Similar to other studies in East and Southern Africa assessing the mental health impact of COVID-19 on women and girls (19, 20), we found that COVID-19 and its response increased anxiety and depression among participants, which has been tied to increased sexual risk behaviors and poor engagement in HIV prevention care (21, 22). Financial hardship due to COVID-19 increased interpersonal violence from partners and changed relationship dynamics, though less frequently among AGYW in our study compared to others (20, 23, 24). While this has been associated with decreased interest and willingness to use PrEP as well as general PrEP adherence in other studies (25, 26), we found little change in HIV prevention interest likely due to the nature of participation in the studies. Economic strains increased frequency of sex work for a few participants, though the total number of transactional sex partners reported decreased overall as found in similar studies among women at risk for HIV (27). AGYW also mentioned decreases in alcohol use since bars were closed and large groups were not able to gather due to COVID-19 restrictions. At the community level, disruptions in schooling and limited social gatherings at local institutions, like church, decreased some participants’ feelings of connection to others while increasing connections to immediate family and partners. These non-pharmaceutical interventions for COVID-19 response have had both positive and negative consequences across Africa (28) At the larger structural level, lockdowns caused disruptions in access to healthcare services, including condoms, which has been experienced across several key populations in Africa (29, 30).

Each of the four countries in which our study population resided—Malawi, South Africa, Uganda, and Zimbabwe—had distinct responses to the COVID-19 pandemic, however, we found few country-level differences in our quantitative or qualitative findings. All four countries made national disaster declarations in the March of 2020, with similar safety precautions instated such as travel bans, school closures, and limitations to large gatherings (31–36). South Africa had some of the most stringent policies, including bans on alcohol and cigarette sales (31, 35). The enforcement and duration of policies varied by country, which likely impacted study participants in different ways. Malawi, for example, saw strong nation-wide resistance to COVID-19 prevention policies including a national court injunction against several measures while Zimbabwe saw alarming increases in police brutality related to lockdown enforcement (31, 32, 34, 36). Policies around mass testing campaigns and mandatory face masks also varied, in part due to differences in the health infrastructure of the four countries. Despite these differences, there did not appear to be significant differences by country on the micro-level impact of COVID-19 on the sexual behavior, HIV prevention interest, general healthcare access, and other HIV risk factors.

Though this study provides rich quantitative and qualitative information about experiences with study product use and perspectives on HIV prevention during the COVID-19 pandemic, our findings should be interpreted in the context of several limitations. First, our participants self-reported data which may have introduced social desirability bias in answers about alcohol use and partner status, as well as demand characteristics for questions about interest in study products and HIV prevention. A second limitation is that our sample only includes a sub-population of trial participants due to the incorporation of COVID-19 questions into study protocols after the trials had started. This convenience sampling means that our study population may not be completely representative of the parent studies. Third, the study population may not be representative of the general population given their participation in the HIV trial setting which raises two concerns: (1) participants in our study may view biomedicine more favorably than those who did not participate, which may have skewed data about HIV interest in a more positive direction; (2) our participants’ access to care was likely higher compared to the general population which may have lowered the impact of COVID-19 on their healthcare experiences during the pandemic. However, we do think that our study populations were equally affected by COVID-19 in domains of interest outside the trial that are still related to HIV vulnerability: such as financial hardship, mental health burden, and changing partner dynamics. Additionally, there is no real-world data on ring use outside of clinical trials, so our study provides important information about the experience of ring users not available elsewhere. The last limitation is that this study was limited to descriptive analyses because everyone in the study was exposed to the pandemic and its response. There was no group that was not exposed to the pandemic for comparison purposes and therefore, we are limited to describing participant’s perceptions of how their attitudes and behaviors shifted during the pandemic.

Overall, the COVID-19 pandemic and its response caused increased strains on many factors related to HIV prevention and vulnerability like economic instability, adverse mental health, increased violence, and decreased access to healthcare services. Future research on the impact of emerging diseases, like COVID-19, on HIV prevention should include community samples outside of structured trials, assess the longitudinal impact after the disease outbreak is controlled, and test ways to alleviate the undue burden experienced by women and girls. In light of the experiences that AGYW, breastfeeding women, pregnant women, and couples in our study faced during the COVID-19 pandemic, we recommend a stronger and more targeted response effort during future disease outbreaks in East and Southern Africa. We propose increased governmental support to ensure the healthcare needs of women and girls are not disrupted and advocate for proactive pandemic training of healthcare workers. Policies, like shelter-in-place, need to consider the complex vulnerabilities of AGYW, and social support programs should specifically target women and girls to ensure that power imbalances and coercion do not further exacerbate inequities.

## 5. Conclusion

Our findings suggest that COVID-19 and its response had a mixed impact on different populations of trial participants in East and Southern Africa. This study indicates that continued access to HIV prevention products is essential, especially for women and girls, who are at a higher risk of HIV infection and disproportionately impacted by COVID-19. More research is needed to better understand the long-term impact of COVID-19 on HIV prevention in community populations and further work is needed to address gender inequities exacerbated by emerging diseases.

## Data Availability

Publicly available datasets were analyzed in this study. Study data are available upon request from the Microbicide Trials Network by submission of a Dataset Request Form available at http://www.mtnstopshiv.org/resources. Interested parties would be able to access these data in the same manner as the authors. The authors did not have any special access privileges that others would not have.
